# Dataset of MAPLE Parameters for Hemoglobin Deposition upon long period gratings

**DOI:** 10.1016/j.dib.2020.105641

**Published:** 2020-04-29

**Authors:** Georgi Dyankov, Tinko Eftimov, Nikola Malinowski, Evdokia Belina, Hristo Kisov, Predrag Mikulic, Wojtek Bock

**Affiliations:** a Institute for Optical Materials and Photonics, Bulgarian Academy of Sciences, 109 Acad. G. Bonchev, Str, Sofia 1113, Bulgaria; b Photonics Research Center, Université du Québec en Outaouais, rue 101 St-Jean Bosco, Gatineau, Québec, J8 X 3G5, Canada

**Keywords:** Matrix-assisted pulsed laser evaporation (MAPLE), Long period gratings around turning point, Biosensors, Hemoglobin, Direct immobilization

## Abstract

Matrix-assisted pulsed laser evaporation (MAPLE) is an alternative and complimentary method to pulsed laser deposition. MAPLE has been demonstrated to be a less harmful approach for transporting and depositing delicate, highly sensitive molecules. Metalloproteins are considered sensitive molecules since their bioactivity is determined not only by their chemical structure but also by conformational changes that can be altered by deposition methods. Here we report a dataset of MAPLE deposition parameters of haemoglobin (Hb) that ensures the retention of its bioactivity. Methods for parameters optimization are also described. The data and analysis should be valuable for researchers interested in application of MAPLE techniques for metalloprotein immobilization since it provides a unique opportunity for direct immobilization. The data presents the results of previously conducted experiments on the basis of which is based the research article entitled “A Highly Efficient Biosensor based on MAPLE Deposited Hemoglobin on LPGs Around Phase Matching Turning Point” [1].

**Specifications Table**

**Table:** 

**Subject**	Biomaterials
**Specific subject area**	Immobilization of biomaterials without built-in matrix for biosensor application
**Type of data**	Table Image Graph Figure
**How data were acquired**	FTIR-BRUKER Vertex 7
	a) AFM- Asylum Research, Oxford Instruments Nd-YAG laser BM Industries 5000 Survey,
**Data format**	Raw Analyzed Filtered
**Parameters for data collection**	Vacuum camera equipped with cooling system and optoelectronic set-up to control deposition parameters
**Description of data collection**	The optoelectronic setup was collecting the data parameters during the deposition process. Custom goniometer equipped with OceanOptics spectrometer for SPR spectra collection. FTIR spectra; AFM scan;
**Data source location**	Institution: Institute of Optical Materials and Technology; City: Sofia, Country: Bulgaria; (N:42.68; E:23.37) Institution: Photonics Research Center, Université du Québec en Outaouais; City: Gatineau; Région: Québec, Country: Canada
**Data accessibility**	Repository name: Mendeley Data https://data.mendeley.com/datasets/zppknydj36/draft?a=46fde1ab-6a98-45f3-9dbb-c2d79db4d04e
**Related research article**	G. Dyankov, T. A. Eftimov, N. Malinowski, E. Belina, H. Kisov, P. Mikulic, W. J. Bock; "A Highly Efficient Biosensor based on MAPLE Deposited Hemoglobin on LPGs Around Phase Matching Turning Point"; Optics and Laser Technology; https://doi.org/10.1016/j.optlastec.2019.105907

**Value of the Data**

The data are а useful resource to achieve successful MAPLE deposition of bioactive Hb film.
The data can serve as a reference for investigators interested in applying the MAPLE technique for biochip development.
The shared data are a dataset of parameters. The optimization of these parameters is complicated since a trade-off between numerous parameters has to be found, with respect to different, even contradictory, optimization criteria. So, we do not claim the shared set of parameters to be ideal. The shared data can be used as a valuable reference point for further optimization of MAPLE technique.
The shared data are specific for Hb deposition. They can be considered as a starting point for MAPLE optimization for deposition of other metalloproteins.

## 1 Data Description

Data reported in Table 1 and Table 2 are summarised MAPLE parameters after the analysis of numerous experiments performed under diverse conditions. Data are collected from protocols of measurements provided by system controlling laser, vacuum camera, cooling system and optoelectronic setup.

**Table 1:** Summary of the Optimal Parameters for Target Substance

Optimal parameters	Value
Target temperature	-40 ^0^C
Downtime at pressure less than 10^−3^Torr	45 min

**Table 2:** Summary of the MAPLE parameters for the successful deposition of hemoglobin (Hb) films

Protein - laser irradiation interaction parameters	Value	Protein - laser irradiation interaction parameters	Value
Wavelength	1.06 μm	Pulse repetition rate	10 Hz
Energy/Pulse	35 mJ	Pulse duration	10 ns
Fluence	160 mJ/cm^2^	Solute concentration	7% wt
Volatile solvent	water	Number of laser shots	45 000

Parameters in Table 1 and Table 2 are complementary – data reported in Table 2 are collected when the experimental conditions correspond to parameters in Table 1.

**Figure 1:**
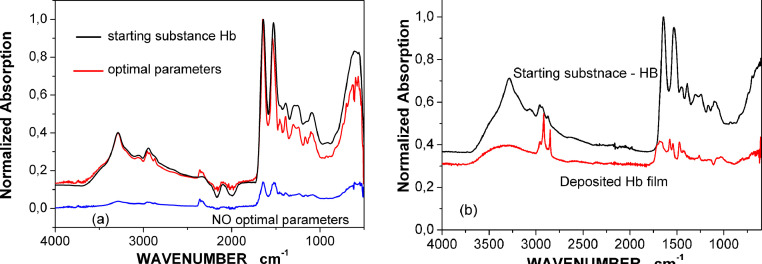
FTIR spectra of a) the starting substance and the substance left in the target holder after deposition and not interacting with the laser irradiation; b) the starting substance and deposited film.

The criteria for the definition „optimal parameters” and “successful deposition” are related to Fig. 1a and Fig. 1b, as explained in the next section. They graphically present the raw data obtained during FTIR spectroscopic measurements. Standard procedure for elimination of CO_2_ absorption around 2000 cm^−1^ was applied. The data format is provided by the software of FTIR spectrometer and no additional data processing was applied.

**Figure 2:**
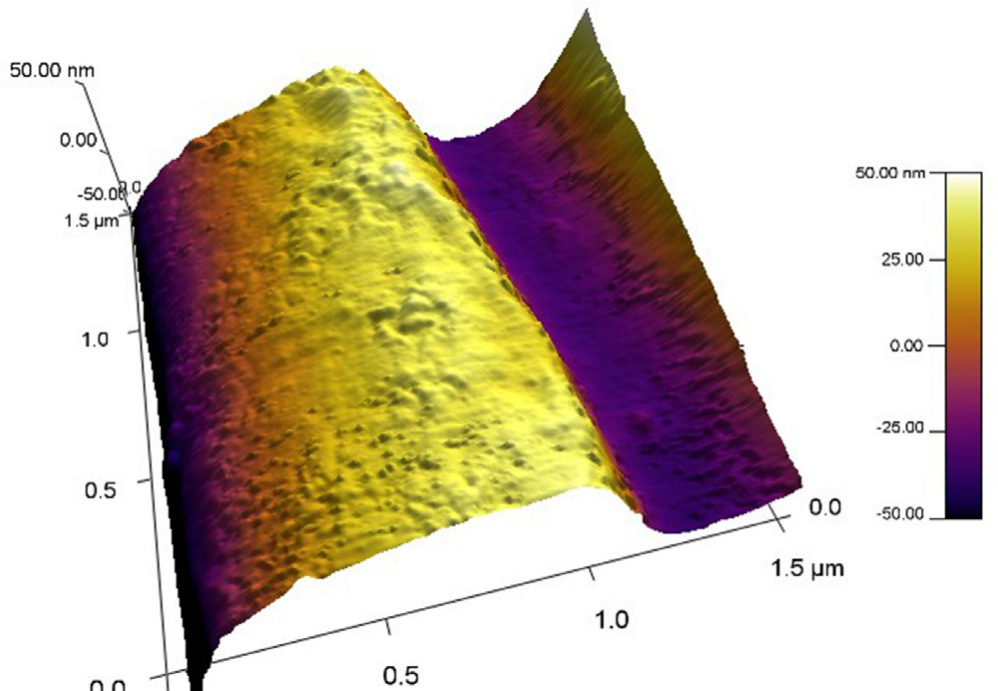
AFM scan of a gilded diffraction grating covered with a 140 nm thick hemoglobin (Hb) film.

Fig. 2 is row data of AFM scan of gilded diffraction grating with deposited Hbfilm. The standard software of AMF instrument was used for getting 3D image.

**Figure 3:**
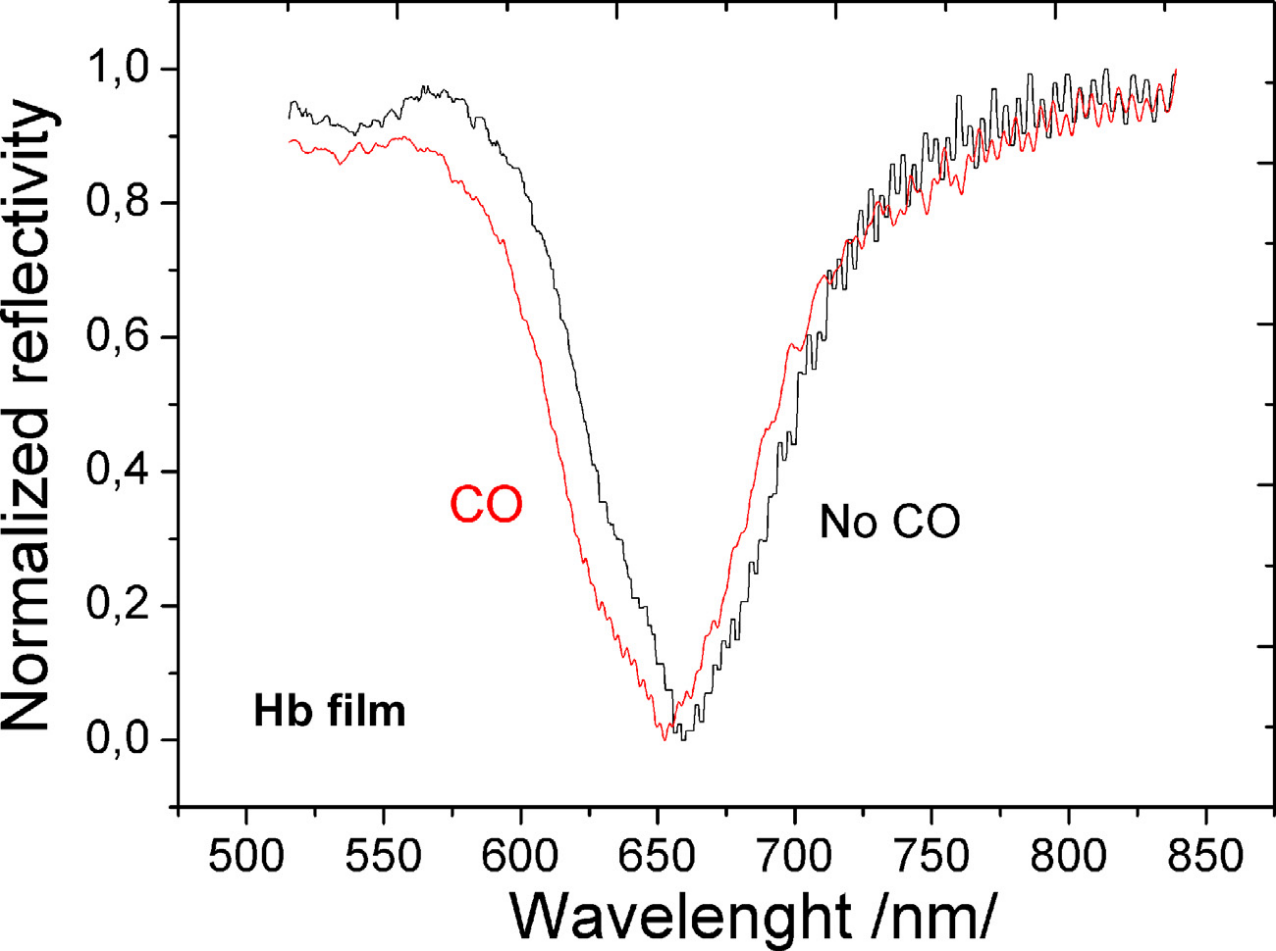
SPR resonance shift as a result of binding of CO to Hb heme group

Fig. 3 is raw data of SPR excited in Hb film deposited on gilded diffraction grating. Data are provided by Ocean Optics Spectrometer in two experiments: i) Hb film not exposed in carbon oxide (CO) - reference; ii) Hb film exposed to CO (500 ppm). The reflectivity is normalized to the S polarization intensity.

## 2 Experimental Design, Materials, and Methods

Some details about parameters of MAPLE technique have been described in [1]. MAPLE has been successfully applied for protein deposition [2,3]. However, it has never been used for the deposition of metalloproteins, such as Hb. Hb is considered to be consisting of sensitive molecules since their bioactivity is determined not only by their chemical structure but also by conformational changes that can be altered by deposition methods. Additionally, OH groups bind to the hydration sites of Hb prevent conformational changes. Then, MAPLE deposition can not only preserve the chemical structure and retain the conformation state of the molecules but also has to minimize the separation of water molecules from Hb molecules, which is inevitable since the process is carried out in vacuum. Establishment of the required parameters is according to criteria that are often controversial. For example, low laser energy is required to preserve the chemical structure and bioactivity, however this results in longer downtime in the vacuum chamber and consequently to separation of the OH groups.

Тo evaluate the role of different parameters in different stages of deposition process, we have considered two type of parameters.

The first type are the process parameters that define the properties of the target substance left in the target holder after MAPLE deposition, but not exposed to laser irradiation. We use the term “optimal/no optimal parameters” to define them. The optimal parameters preserve the properties of the targeted substance, are reported in Tabl.1. The properties of the substance are evaluated via FTIR spectroscopy. The spectroscopic row data as graphics are presented in Fig. 1a and demonstrate that MAPLE deposition at the optimal parameters does not destroy protein remaining in the target holder. The comparison is with the started substance Hb.

If the films are deposited at optimal parameters, their properties are defined by the second type of parameters, related to the interaction between Hb molecules and laser irradiation. The impact of these parameters is evaluated by FTIR spectroscopy of deposited films. Fig. 1b shows graphically the raw data of FTIR measurements of film deposited (on Si substrate) at parameters listed in Tabl.2.Obviously, the main characteristic bands are expressed; however, some are missing.

The question is whether the parameters listed in Tabl.2 influence the bioactivity of the deposited film in spite of the observed differences in FTIR spectra. The bioactivity of MAPLE-deposited films was measured by means of surface plasmon resonance (SPR). For this purpose, the Hb film was deposited under the conditions, listed in Table 1 and Table 2, upon a gilded diffraction grating. Fig. 3 shows such a grating. Details about the SPR excitation technique can be found in [4].

We use the term “successful deposition/immobilization” to refer to MAPLE processes that produce a film with experimentally proven bioactivity. This is accomplished by checking the vitality of various functional groups of Hb molecules. For this purpose, the bonding of carbon monoxide (CO), carbon dioxide (CO_2_), and nitric oxide (NO) to the corresponding functional groups of Hb/Mb were registered by SPR.

Fig. 3 shows the spectral shift of the plasmon resonance that resulted from the binding reaction between the heme group of hemoglobin and carbon monoxide (500 ppm). We use this reaction to illustrate the bioactivity of the deposited film since the heme group is the most sensitive to deposition parameters. For the MAPLE-deposited metalloprotein films, binding to the amine and thiol groups was observed via corresponding spectral shifts in SPR for CO_2_ (1000 ppm) and NO (700 ppb). A registered spectral shift of about 5–8 nm is a testament to the activity of deposited films and is an integral criterion ensuring the efficiency of established dataset of parameters for successful immobilization, listed in Table 1 and Table 2.

## Credit Author Statement

The individual contributions of the authors this joint research work are as follows:

1 **Georgi Dyankov**: conceptualisation, Writing original draft on MAPL technology, formal analysis, methodology, supervision on MAPLE technology, fund acquisition, project administration
2 **Tinko Eftimov**: conceptualisation, writing original draft on LPG sensing, supervision on LPG measurements, data analysis, review and editing, project administration
3 **Nikola Malinowski**: conceptualisation and methodology, data analysis, resources
4 **Evdokia Belina**: formal analysis, investigation, validation, visualisation, data curation
5 **Hristo Kisov**: investigation, data curation
6 **Predrag Mikulic**: validation, LPG fabricaiton
7 **Wojtek Bock**: review and editing, fund acquisition, project administration

## Declaration of Competing Interest

The authors declare that they have no known competing financial interests or personal relationships which have, or could be perceived to have, influenced the work reported in this article.

## Appendix Supplementary materials

Image, application 1
